# Notes and Notices

**Published:** 1891-01

**Authors:** 


### NOTES AND NOTICES.

Woman's Homœopathic Hospital.—During October eleven patients were admitted to the Woman's Homœopathic Hospital at Susquehanna Avenue and Twentieth Street. There were twenty-five patients in the hospital during the month and nine were discharged. There were five obstetrical cases and two surgical operations. In the dispensary one hundred and thirty new patients were treated, of whom thirty were eye and ear cases, forty-five were surgical cases, six were dental cases, fifty-two were gynæcological cases, and two hundred and thirty-two were medical cases. The prescriptions prepared in the dispensary numbered three hundred and seventy-five. Seventeen patients were visited in homes and forty-six out-visits were made.

Education in Homœopathy.—The Philadelphia Post-Graduate School of Homœopathics has applied to the Common Pleas for incorporation. The purpose of the organization is to educate persons holding diplomas of any reputable medical college of any school of medicine in the United States or elsewhere in the philosophy and practice of homœopathic medicine, to matriculate students and confer the degree of Master of Homœopathics and to issue diplomas in testimony of the same. The corporators are: John Pitcairn, Theodore P. Matthews, Wm. A. Drown Pierce, M. D., Wm. H. A. Fritz, M. D., Wm. F. Kaercher, James T. Kent, M. D., Milton Powel, M. D., Arthur G. Allan, M. D., and Robert Bruce Johnstone, M. D., of Philadelphia; Franklin Powel, M. D., of Chester; Robert Farley, M. D., of Phœnixville.—*Phila. Ledger.*

The establishment of a post-graduate school for the teaching of pure Hahnemannian Homœopathy, with teachers of undoubted loyality to its principles, is a step in the right direction, and certainly fills a much-needed want of our school. All success to all such endeavors!

The Open Court Publishing Company, of Chicago, will publish immediately, in two handsomely bound and printed volumes, a new, authorized translation of Gustav Freytag's well-known novel, *The Lost Manuscript.* This is regarded by critics as the most charming of the famous German writer's works.

